# *HER2* copy number as predictor of disease-free survival in HER2-positive resectable gastric adenocarcinoma

**DOI:** 10.1007/s00432-021-03522-9

**Published:** 2021-02-04

**Authors:** Zimin Liu, Mingpeng Shi, Xiaoxiao Li, Shanai Song, Ning Liu, Haiwei Du, Junyi Ye, Haiyan Li, Zhou Zhang, Lu Zhang

**Affiliations:** 1 Oncology Department, The Affiliated Hospital of Qingdao University, Qingdao, 266000 China; 2 Burning Rock Biotech, Guangzhou, China; 3 Operating Room of Neurosurgery, The Affiliated Hospital of Qingdao University, Qingdao, 266000 China

**Keywords:** *HER2*, Copy number, Resectable gastric adenocarcinoma, Survival

## Abstract

**Purpose:**

The identification of HER2 overexpression in a subset of gastric adenocarcinoma (GA) patients represents a significant step forward in unveiling the molecular complexity of this disease. The predictive and prognostic value of *HER2* amplification in advanced HER2 inhibitor-treated GA patients has been investigated. However, its predictive value in resectable patients remains elusive.

**Methods:**

We enrolled 98 treatment-naïve resectable Chinese GA patients with HER2 overexpression assessed using IHC. Capture-based targeted sequencing using a panel consisting of 41 gastrointestinal cancer-related genes was performed on tumor tissues. Furthermore, we also investigated the correlation between *HER2* copy number (CN) and survival outcomes.

**Results:**

Of the 98 HER2-overexpressed patients, 90 had *HER2* CN amplification assessed using next-generation sequencing, achieving 92% concordance. The most commonly seen concurrent mutations were occurring in *TP53*, *EGFR* and *PIK3CA*. We found *HER2* CN as a continuous variable was an independent predictor associated with DFS (*p* = 0.029). Our study revealed *HER2* CN-high patients showed a trend of intestinal-type GA predominant (*p* = 0.075) and older age (*p* = 0.07). The median *HER2* CN was 15.34, which was used to divide the cohort into CN-high and CN-low groups. Patients with high *HER2* CN had a significantly shorter DFS than patients with low *HER2* CN (*p* = 0.002). Furthermore, *HER2* CN as a categorical variable was also an independent predictor associated with DFS in patients.

**Conclusion:**

We elucidated the mutation spectrum of HER2-positive resectable Chinese GA patients and the association between *HER2* CN and DFS. Our work revealed *HER2* CN as an independent risk factor predicted unfavorable prognosis in HER2-positive GA patients and allowed us to further stratify HER2-positive resectable GA patients for disease management.

## Introduction

Gastric cancer (GC) is the fifth most frequently diagnosed cancer and the third leading cause of cancer death in the world (Bray et al. 2018). Despite a worldwide decline in the incidence of GC, the mortality rate due to GC remains high (Siegel et al. 2020) because the majority of patients are diagnosed at an advanced stage and patients undergoing curative tumor resection for early-stage-GC frequently experience recurrence and or progression to metastatic disease (Hohenberger and Gretschel 2003; Van Cutsem et al. 2016).

*HER2* (known as *ERBB2,* human epidermal growth factor receptor 2) represents the first molecular target for GC patients. It encodes human epidermal growth factor receptor 2, a 185 KD transmembrane glycoprotein receptor with intracellular tyrosine kinase activity, and is activated by homodimerization or heterodimerization with other ERBB receptors including HER3 (Coussens et al. 1985; Hsieh and Moasser 2007; King et al. 1985; Ma et al. 2014). It is well known that 8–30% gastric adenocarcinomas (GA) show HER2 overexpression or gene amplification (Aditi et al. 2016; Bang et al. 2010; Cordero-García et al. 2019; Qiu et al. 2017; Shen et al. 2016; Shitara et al. 2013). Overexpression of HER2 drives tumorigenesis, resulting in activated oncogenic downstream signaling, such as PI3K/Akt/mTOR and MAPK, promoting cellular proliferation, survival and angiogenesis in vitro and in vivo (Hynes and Stern 1994; Moasser 2007; Yarden and Sliwkowski 2001). Based on that observation, HER2 has been targeted for cancer treatment.

Several studies reported that HER2 overexpression predicts unfavorable prognosis in unresectable GC patients (Mizutani et al. 1993). HER2 is also a predictive marker for survival benefit in advanced GC patients treated with trastuzumab plus chemotherapy. The ToGA (Trastuzumab for Gastric Cancer) trial reveals that HER2*-* positive patients experienced longer overall survival (OS) after treated with trastuzumab plus chemotherapy compared with HER2*-*positive patients treated with chemotherapy alone (Bang et al. 2010). However, the prognostic value of HER2 overexpression or gene amplification in resectable GA patients remains controversial. Some studies reported HER2 overexpression predicts unfavorable prognosis (Garcia et al. 2003; Kurokawa et al. 2015), but other studies failed to find an association between HER2 expression level and prognosis (Kim et al. 2019; Shen et al. 2016). In the present work, *HER2* amplification was assessed by targeted next-generation sequencing (NGS) using a panel consisting of 41 gastrointestinal cancer-related genes, and the predictive and prognostic value of *HER2* amplification in patients with HER2-positive resectable GA was investigated.

## Methods

### Patients and specimens

A total of 98 consecutive patients with any stage of primary GA and positive HER2 expression (+++) measured by immunohistochemistry staining (IHC) underwent gastrectomy at the Affiliated Hospital of the Qingdao University were between March 2013 and December 2017 were retrospectively recruited. Ninety-two stage I-III patients received curative gastrectomy and 6 stage IV patients received palliative gastrectomy. This study was approved by the Ethics Committee of the Affiliated Hospital of Qingdao University and informed consent was obtained from all patients. For each tumor, formalin-fixed, paraffin-embedded (FFPE) tumor tissue was obtained after surgery, 78.6% samples (77 of 98) had a tumor content ≥ 50% (ranged from 50 to 80%) and 21.4% samples (21 of 98) were lower than 50% (ranged from 10 to 40%). Disease-free survival (DFS) was defined as the interval from the date of surgery to the date of first locoregional tumor recurrence, distant metastasis or death. Overall survival (OS) was defined as the interval from the date of surgery to the date of death. Patients without an event were censored at the time of the last visit in follow up.

### Immunohistochemistry staining and results scoring

IHC staining was performed on 4 μm-thick FFPE tissue sections according to the manufacturer’s instructions. Briefly, after deparaffinization, heat-induced antigen retrieval and endogenous peroxidase blocking, sections were incubated with ready-to-use anti-HER2 rabbit monoclonal primary antibody (clone 4B5, Ventana, Roche diagnostics, Indianapolis, IN, USA). Staining was visualized using the Ventana ultraView Universal DAB Detection (Ventana, Roche diagnostics, Indianapolis, IN, USA). Slides were lightly counterstained with hematoxylin. Positive and negative controls were included in each assay.

IHC staining results of HER2 expression was scored based on the intensity of membranous staining in tumor cells. HER2 IHC were evaluated according to Hofmann’s criteria for gastric cancer (Hofmann et al. 2008), which is scored as 0 (negative), 1 + (negative), 2 + (equivocal) or 3 + (positive). Those samples with a score of 3 + were considered HER2-positive and were subjected to further capture-based targeted sequencing. IHC scoring was performed by two independent pathologists and a final score was achieved by consensus.

### Capture-based targeted sequencing

FFPE DNA was extracted and fragmented followed by end repair, phosphorylation, dA addition, and adaptor ligation for library construction. Then, libraries enriched with a targeted next-generation sequencing panel (Burning Rock Biotech Ltd., Guangzhou, China) consisting of 41 gastrointestinal cancer-related genes (as Table S1 shown) were sequenced on Illumina NextSeq 500 (Illumina, Inc., San Diego, CA, USA) with pair-end reads.

### Sequencing data processing and prediction of copy number variations

The sequencing reads were aligned to the human genome (hg19) using Burrows-Wheeler Aligner 0.7.10. Genome Analysis Toolkit 3.2 (Broad Institute, Cambridge, MA) was used for base quality score recalibration. Mutations were filtered against common single nucleotide polymorphisms found in 1000 Genomes ExAC, dbSNP, ESP6500SI-V2, and ClinVar databases. Variant calling was performed by using VarScan. Variants were annotated with ANNOVAR and SnpEff v3.6. All tumor samples showed adequate coverage, with an average sequencing depth of 1280 $\times$. Identification of copy number variations (CNVs, amplifications and deletions) was executed as a previous description.

### Statistical analysis

Differences in two-groups were accessed by chi-square test for categorical data and *t*-test for continuous variable. Kaplan–Meier curves were compared by using the log-rank test for survival analyses. Univariate and multivariate analyses with the Cox proportional hazards model were applied. p < 0.05 was considered as statistically significant. All statistical analyses were performed in SPSS 22.0 (IBM, Armonk, NY, USA) and R language (version R 3.3.3., https://www.r-project.org/).

## Results

### Patients characteristics

A total of 98 (81 males and 17 females) treatment-naïve resectable Chinese GA patients with HER2 overexpression assessed using IHC with stage I-IV GA were enrolled in our study. The median age of the cohort was 64 years (ranged from 35 to 87 years). There were 12 stage I cases (12.2%), 33 stage II cases (33.7%), 47 stage III cases (48.0%) and 6 stage IV cases (6.1%). For histological grade, 3 patients (3.1%) had well differentiated GA, 41 (41.8%) had moderately differentiated GA, 53 (54.1%) had poorly differentiated GA and the status of histological grade of the remaining 1 patient (1.0%) was unknown. According to the Lauren’s classification, this cohort had 62 intestinal-type cases (63.3%), 12 diffuse-type cases (12.2%), and 24 mixed-type cases (24.5%). Furthermore, 59 patients (60.2%) were positive for perineural invasion, and 39 (39.8%) were negative. There were 60 patients (61.2%) with lymphatic/vascular invasion and 38 (38.2%) without. Eighty-two patients (83.7%) had a positive ulcer, 14 patients (14.3%) had a negative ulcer and the status of ulcer findings in remaining 2 patients (2.0%) was unknown. Fifteen patients (15.3%) had proximal GA, 40 (40.8%) had middle GA, 40 (40.8%) had distal GA and the status of tumor location in remaining 3 patients (3.1%) was unknown. The clinical characteristics of the cohort were summarized in Table 1. In addition, a majority of stage II-IV patients received adjuvant chemotherapy.

**Table 1:** Clinical baseline of patients with resectable HER2-positive GA

Characteristics	*n* (%)
Median age (range), years	64 (35–87)
*Gender*	
Female	17 (17.3%)
Male	81 (82.7%)
*Stage*	
I	12 (12.2%)
II	33 (33.7%)
III	47 (48.0%)
IV	6 (6.1%)
*Histological grade*	
Well differentiated	3 (3.1%)
Moderately differentiated	41 (41.8%)
Poorly differentiated	53 (54.1%)
Unknown	1 (1.0%)
*Lauren’s classification*	
Intestinal type	62 (63.3%)
Diffuse type	12 (12.2%)
Mixed type	24 (24.5%)
*Perineural invasion*	
Positive	59 (60.2%)
Negative	39 (39.8%)
*Lymphatic/venous invasion*	
Positive	60 (61.2%)
Negative	38 (38.8%)
*Ulcer findings*	
Positive	82 (83.7%)
Negative	14 (14.3%)
Unknown	2 (2.0%)
*Tumor location*	
Proximal	15 (15.3%)
Middle	40 (40.8%)
Distal	40 (40.8%)
Unknown	3 (3.1%)

*HER2* human epidermal growth factor receptor 2,* GA* gastric adenocarcinoma,* TNM* tumor, node, metastasis

### Mutation profiling in tumor samples

**Fig. 1:**
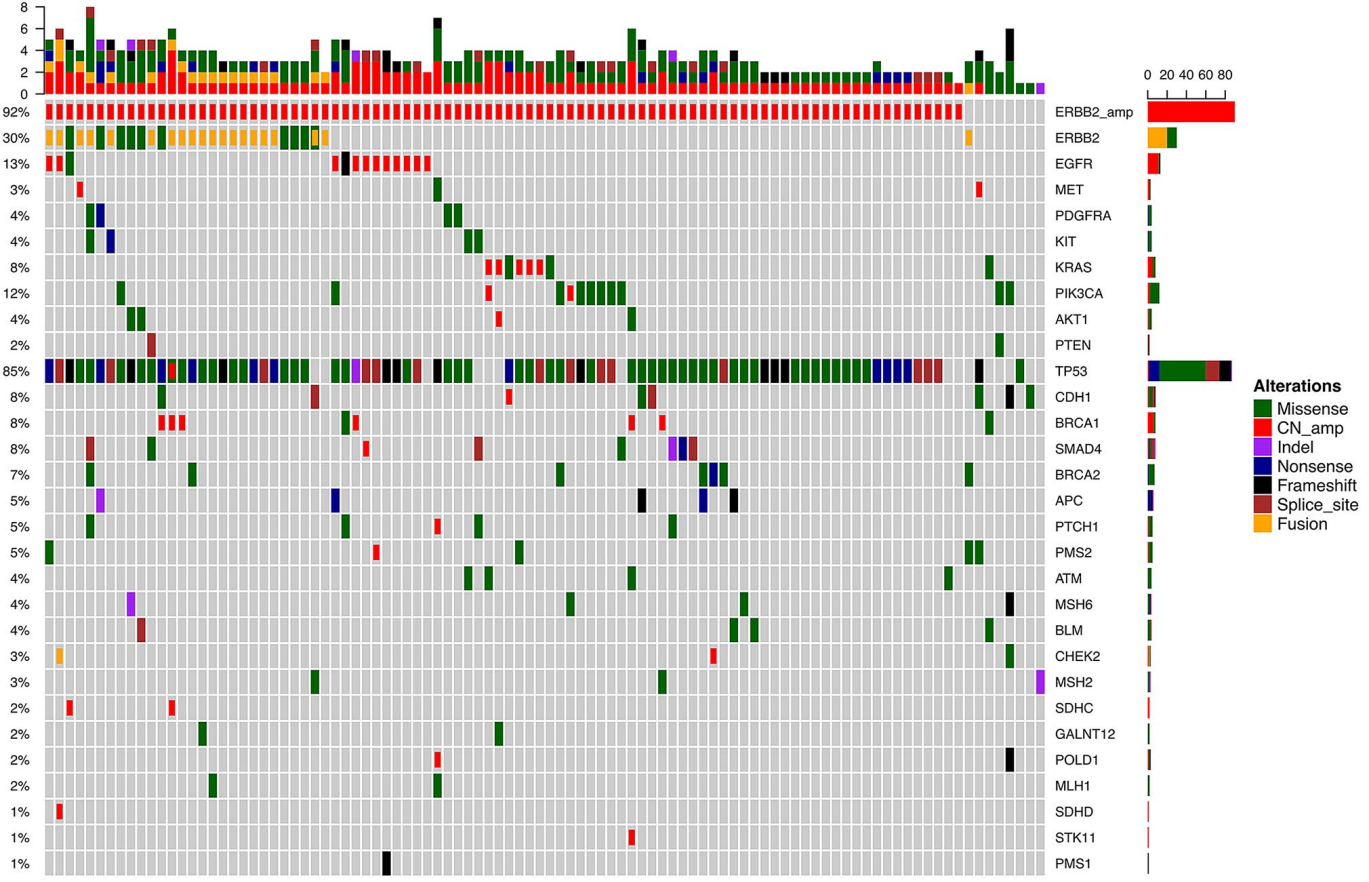
Heatmap of patients harboring genetic mutations in tumor samples retrieved from targeted next-generation sequencing panel consisting of 41 gastrointestinal cancer-related genes

Ninety-eight HER2-positive samples assessed using IHC analysis were subjected to targeted sequencing**.** Collectively, 344 genetic alterations spanning 29 genes were identified, including 186 SNVs, 25 rearrangements, 5 indels and 128 copy number amplifications (CNAs). All patients except 2 had at least one concurrent mutation. The most commonly seen concurrent mutations were occurring in *TP53*, *EGFR* and *PIK3CA*, occurring in 84.7% (*n* = 83), 13.3% (*n* = 13) and 12.2% (*n* = 12) patients, respectively. Eighteen patients (18.4%) had mutations in *PIK3CA, AKT1* or *PTEN*. None of the patients carried *NRAS* or *BRAF* mutations. Ninety patients had *HER2* amplification defined by NGS, resulting in a concordance of 92%. Ten patients had concurrent *HER2* missense mutations, including 4 with S310X. In addition to *HER2* amplification, 16 genes were concomitantly co-amplified. Six co-amplified genes occurred in more than one patient, including *EGFR* (*n* = 11), *BRAC1* (*n* = 6), *KRAS* (*n* = 5), *PIK3CA* (*n* = 2), *SDHC* (*n* = 2), *MET* (*n* = 2) (Fig. 1).

### The association of *HER2* copy number with clinicopathologic features

**Fig. 2:**
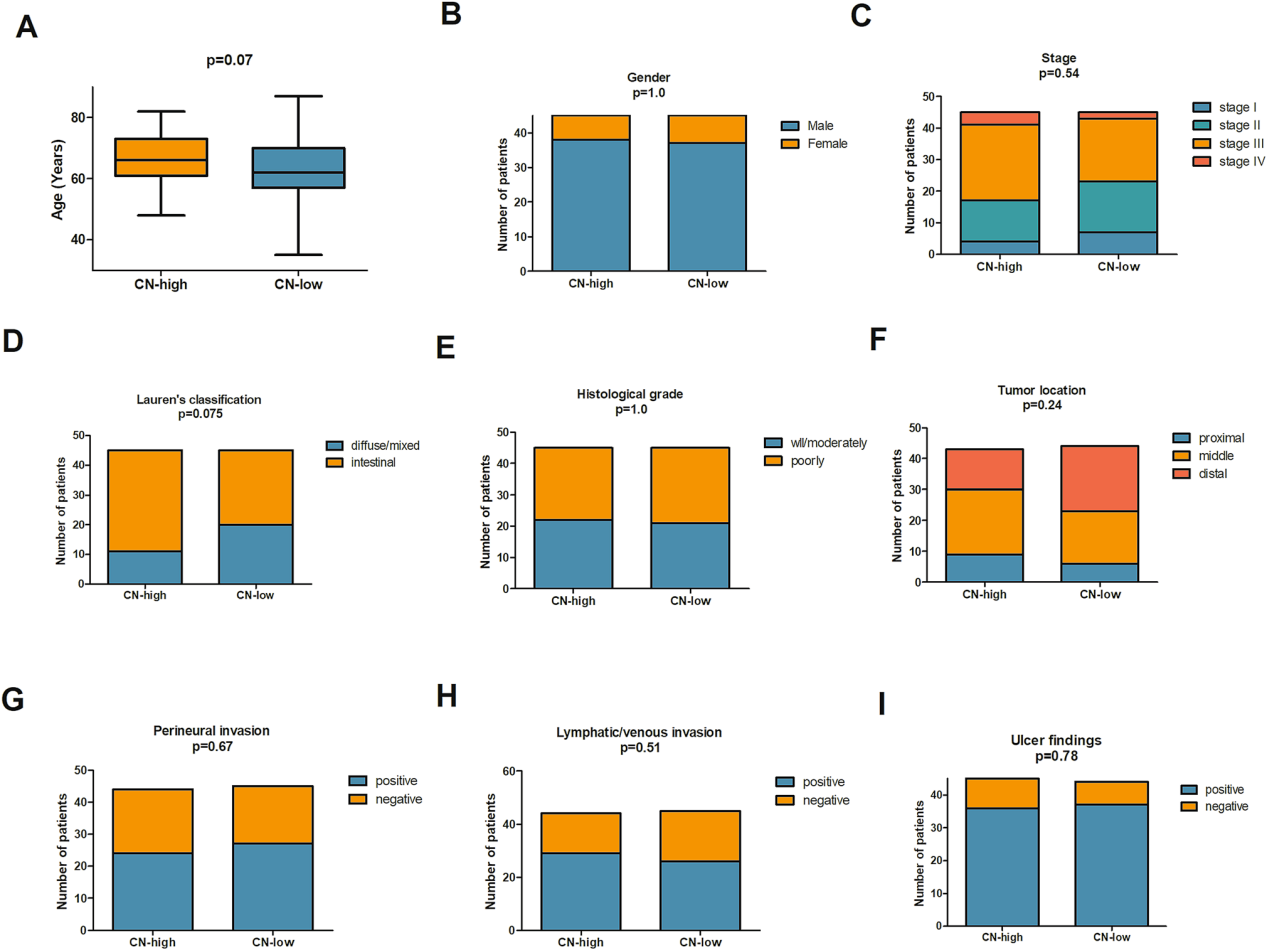
Correlation of *HER2* copy number with clinicopathologic features in HER2-positive resectable GA patients. (**a**), age; (**b**), gender; (**c**), stage; (**d**), Lauren’s classification; (**e**), histological grade; (**f**), tumor location; (**g**), perineural invasion; (**h**), lymphatic/venous invasion; (**i**), ulcer findings.* GA* gastric adenocarcinoma,* CN* copy number, *HER2* human epidermal growth factor receptor 2

*HER2* copy number (CN) gain was observed in 90 GA patients (90/98) with a median CN of 15.34 (ranged from 2.67 to 116.27, Fig. S1), which was used to divide the cohort into CN-high (*n* = 45) and CN-low groups (*n* = 45). Next, we evaluated the association between *HER2* CN and several clinical characteristics. Patients with high *HER2* CN showed a trend of older compared with patients with low CN (*p* = 0.07, Fig. 2a). According to Lauren’s classification, *HER2* CN-high group showed a trend of association with intestinal-type GA predominant (*p* = 0.075, Fig. 2d). No significant difference of gender, stage, histological grade, tumor location, perineural invasion, lymphatic/venous invasion and ulcer findings was observed between CN-high and CN-low group (Fig. 2).

### The associations between *HER2* copy number and survival outcomes

The associations between *HER2* CN and survival outcomes including DFS and OS in HER2-positive resectable GA patients with stage I-III were evaluated. Forty-nine patients had available *HER2* CN and DFS. We investigated whether *HER2* CN as a continuous variable was an independent predictor in HER2-positive resectable GA patients. Our work demonstrated that *HER2* CN as a continuous variable was significantly associated with DFS in the univariable Cox proportional hazards regression model (HR: 1.02, 95% CI 1.00–1.04, *p* = 0.025) and it retained the significant association with DFS in the multivariate Cox proportional hazards regression model (HR: 1.05, 95% CI 1.01–1.09, *p* = 0.029) (Table 2).

**Table 2:** *HER2* CN as a continuous variable was the independent predictor associated with DFS

Characteristics	Univariable analysis		Multivariable analysis	
	HR (95% CI)	*p*-value	HR (95% CI)	*p*-value
Gender Male vs. female	1.47 (0.18–11.74)	0.719	2.39 (0.20–28.96)	0.494
Age ≥ 60 vs. < 60 years	2.81 (0.58–13.63)	0.198	6.67 (0.55–80.91)	0.136
Stage III vs. I/II	3.59 (0.76–16.98)	0.108	0.98 (0.08–11.79)	0.985
Histological grade poorly vs. well/moderately	3.18 (0.67–15.03)	0.145	9.41 (0.33–267.23)	0.189
Lauren’s classification intestinal vs. diffuse/mixed	0.62(0.17–2.31)	0.474	1.15 (0.12–10.72)	0.900
Perineural invasion positive vs. negative	1.54 (0.38–6.15)	0.543	0.37 (0.05–2.69)	0.326
Lymphatic/venous invasion positive vs. negative	2.05 (0.52–8.07)	0.303	7.24 (0.84–62.56)	0.072
Ulcer findings positive vs. negative	2.09 (0.26–16.73)	0.487	7.60 (0.24–239.13)	0.249
Tumor location distal vs. middle/proximal	0.57 (0.12–2.75)	0.483	0.78 (0.09–6.62)	0.823
*HER2* CN	1.02 (1.00–1.04)	0.025	1.05 (1.01–1.09)	0.029

*DFS* disease-free survival,* HR* hazard ratio,* CI* confidence interval,* CN* copy number, *HER2*: human epidermal growth factor receptor 2

**Fig. 3:**
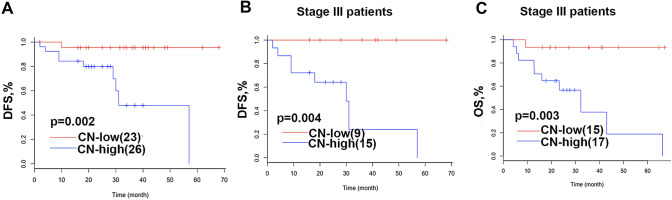
Kaplan–Meier curves of *HER2* CN-high and CN-low patients for DFS and OS. **a** Kaplan–Meier curves of *HER2* CN-high and CN-low patients for DFS in stage I-III patients; **b**, Kaplan–Meier curves of *HER2* CN-high and CN-low patients for DFS in stage III patients; **c** Kaplan–Meier curves of *HER2* CN-high and CN-low patients for OS in stage III patients.* DFS* disease-free survival, * OS* overall survival, * CN* copy number, *HER2* human epidermal growth factor receptor 2

**Fig. 4:**
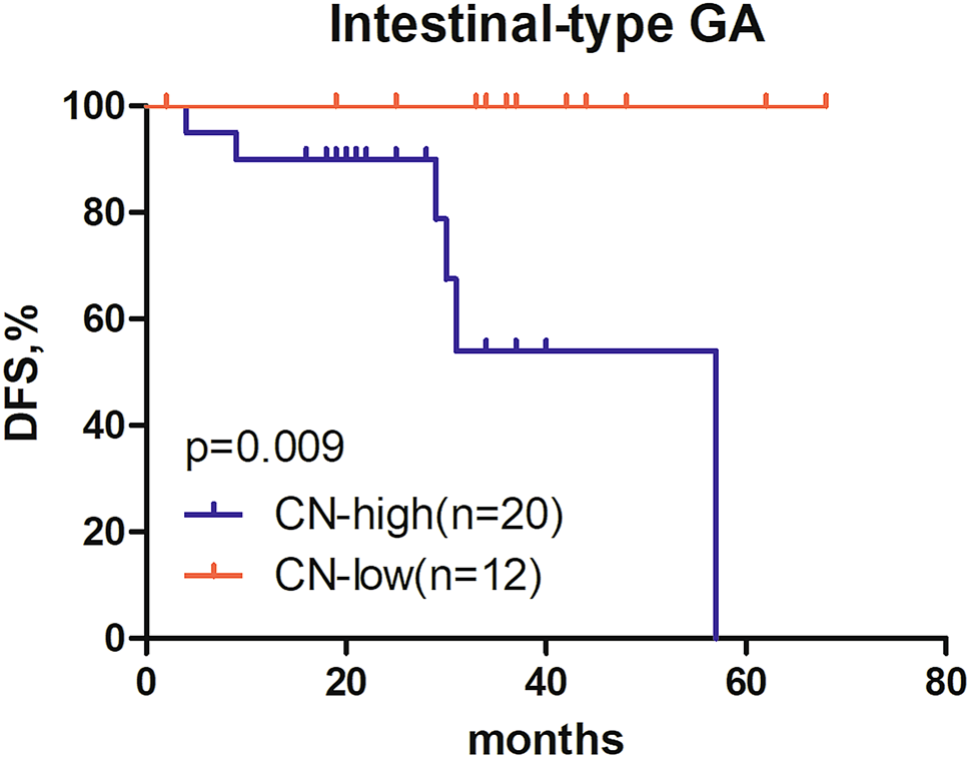
Kaplan–Meier curves of *HER2* CN-high and CN-low patients for DFS in patients with intestinal-type GA. DFS: disease-free survival. CN: copy number; *HER2*: human epidermal growth factor receptor 2

Next, the associations between *HER2* CN as a categorical variable and survival outcomes in patients were also investigated. Stage distribution in CN high and low groups was comparable (*p* = 0.54). Our study revealed that DFS of patients with CN-high (*n* = 26) was significantly shorter than that of those with CN-low (*n* = 23; 31 months vs. not reached; *p* = 0.002; Fig. 3a). A detailed survival analysis was done on patients with stage III disease due to the completeness of survival data. Our observation of shorter DFS in patients with higher CN was confirmed in this subset of patients. Of 24 stage III patients with available DFS, patients with CN-high (*n* = 15) had significantly shorter DFS than those with CN-low (*n* = 9; 31 months *vs*. not reached; *p* = 0.004; Fig. 3b). Of 32 patients with the available OS, patients with CN-high (*n* = 17) also had significantly shorter OS than those with CN-low (*n* = 15; 32 months *vs*. not reached; *p* = 0.003; Fig. 3c). Furthermore, stratified analyses were performed to explore the correlations of *HER2* CN with survival outcomes in intestinal-type GA patients due to the fact that a majority of patients was diagnosed with intestinal-type GA in our work. Of 32 intestinal-type GA patients with available DFS, patients with CN-high (*n* = 20) had significantly shorter DFS than those with CN-low (*n* = 12; 57 months *vs*. not reached; *p* = 0.009; Fig. 4). Collectively, our analyses revealed that patients with high CN are associated with shorter DFS. Furthermore, stage III patients with high CN are associated with shorter OS.

In order to evaluate whether *HER2* CN as a categorical variable was the independent predictor associated with DFS in HER2-positve resectable GA patients, univariable Cox proportional hazards regression model analysis was performed. In the univariable Cox proportional hazards regression model (Table 2), only *HER2* level was associated with DFS (HR: 11.7, 95% CI 1.45–94.15, *p* = 0.021). Furthermore, in the multivariate Cox proportional hazards regression model that included gender, age, stage, histological grade, Lauren’s classification, perineural invasion, lymphatic/venous invasion, ulcer findings, tumor location and *HER2* level, *HER2* level retained the significant association with DFS (HR: 32.6, 95% CI 2.53–419.64, *p* = 0.008) (Table S2). Collectively, *HER2* level either as a continuous variable or as a categorical variable was the independent predictor associated with DFS in HER2-positve resectable GA patients.

### Correlations between concomitant alterations and clinical outcomes

The correlations between concomitant alterations including *TP53, EGFR* and *PIK3CA* mutations and clinical outcomes in HER2-positive resectable GA patients were also investigated. *HER2* CN was comparable in patients with and without *TP53* mutations, in patients with and without *EGFR* mutations, in patients with and without *PIK3CA* mutations (Fig. S2A–S2C). Of 49 GA patients with available DFS, DFS of patients with *TP53* mutations (*n* = 46) and that of patients without *TP53* mutations (*n* = 3; 57 months vs. not reached; *p* = 0.42; Figure S3A) was comparable; DFS of patients with *EGFR* mutations (*n* = 7) and that of patients without *EFGR* mutations (*n* = 42; 57 months vs. not reached; *p* = 0.29; Figure S3B) was comparable; DFS of patients with *PIK3CA* mutations (*n* = 6) and that of patients without *PIK3CA* mutations (*n* = 43; 57 months vs. not reached; *p* = 0.66; Fig. S3C) was also comparable. Collectively, our analyses revealed that concurrent mutations in *TP53*, *PI3KCA* and *EGFR* had no impact on DFS.

## Discussion

The predictive value of HER2/*HER2* levels in HER2 inhibitor-treated advanced GA patients has been investigated. However, its predictive value in resectable patients remains controversial. In this study, NGS was used to evaluate *HER2* CN in HER2-positve resectable GA patients. Our work revealed that *HER2* CN either as a categorical variable or as a continuous variable was the independent risk factor associated with DFS in HER2-positve resectable GA patients.

NGS has become a useful tool for genomic profiling. We confirmed the performance in evaluating CNV using NGS, achieving a concordance of 92% between NGS and IHC for evaluating HER2 CN in GA tumor samples, which were consistent with previous studies (Cenaj et al. 2019; Pfarr et al. 2017; Ross et al. 2017; Su et al. 2017; Yeh et al. 2019). In our work, median *HER2* CN accessed using NGS was 15.34 (ranged from 2.67 to 116.27) in HER2-positive resectable GA patients with a majority of them (94.4%) having a *HER2* CN less than 50, which was in an agreement with previous studies that median *HER2* CN accessed by FISH is 11.9 (ranged from 3.30 to 43.80) and it accessed by droplet digital polymerase chain reaction is about 14 (ranged from 3 to 50) in HER2-positive GC patients (Gomez-Martin et al. 2013; Kim et al. 2020). Our study revealed that intestinal-type GA is more likely to harbor a high *HER2* CN (*p* = 0.075), which was consistent with the previous findings that HER2 overexpression is associated with intestinal-type GC (Kataoka et al. 2013; Kim et al. 2019; Kurokawa et al. 2015).

Previous reports revealed that HER2 overexpression or gene amplification correlated with unfavorable prognosis in resectable GA patients (Kurokawa et al. 2015; Otsu et al. 2015). A similar result was observed in our study that *HER2* CN-high predicted shorter DFS in patients with HER2-positive resectable GA. Although our and prior studies reported that HER2 overexpression led to a poor outcome; in contrast, others reported that HER2 expression level was not related to prognosis in resectable GC (Kataoka et al. 2013; Kim et al. 2019; Shen et al. 2016). The controversial results may be attributed to several factors. First, different methods for assessing HER2/*HER2* level were used across studies, including but not limited IHC, fluorescence in situ hybridization (FISH) and NGS. IHC and FISH constitute the current gold standard for HER2 assessment. Tumor samples with HER2 IHC 3 + or IHC 2 + /FISH + are identified as HER2-positive expression (Hofmann et al. 2008; Muller et al. 2015). There are limitations associated with IHC and FISH for evaluating HER2 overexpression or *HER2* amplification, including variation among different antibody sensitivities and specificities, interobserver variabilities (Koopman et al. 2018; Layfield et al. 2016; Sheffield et al. 2014). Second, different studies included different study population. For example, HER2/*HER2* status is not correlated with prognosis in a cohort consisting of stage I-IV resectable GC patients; however, a negative correlation is apparent in stage III/IV patients (Kataoka et al. 2013). Third, tumor heterogeneity is more frequently seen in GC (Hofmann et al. 2008). The discordance of HER2 overexpression between different areas of the same primary tumor occurred in 14.5% of GC patients, which may lead to an inconsistent assessment of HER2 status (Kim et al. 2011). In addition, the median *HER2* CN of 15.34 as the cutoff for predicting longer DFS was identified in our work. Evidence suggest that *HER2* CN of 10 and 9.5 is the optimal cutoff for predicting OS longer than 12 months and for OS longer than 16 months in HER2-positive advanced GC patients treated with trastuzumab, respectively (Gomez-Martin et al. 2013). The cutoff value of *HER2* CN or predicting longer DFS in HER2-positive resectable GA patients needed to be validated in larger cohorts.

The incidence rate of GC remains high in East Asian countries, primarily attributing to the high prevalence of *Helicobacter pylori* (*H. pylori*) infection; in contrast, it is relatively rare in Western countries (Ferlay et al. 2013; Sugano 2019). Previous study reveals that *H. pylori* infection inducing the aberrant activation-induced cytidine deaminase (AID) via NF-κB activation, results in mutation accumulation in the gastric mucosa during *H. pylori*-associated gastric carcinogenesis (Maeda et al. 2017; Matsumoto et al. 2007). Yoo and colleagues have reported that HER2 overexpression is more prevalent in *H. pylori*-positive GC (Yoo et al. 2014). The abovementioned findings prompt us to speculate that the HER2/*HER2* levels might be different between East Asian and Western population. Several studies have reported the correlation between prognosis and HER2/*HER2* levels in East Asian population (Kataoka et al. 2013; Kim et al. 2019; Kurokawa et al. 2015; Shen et al. 2016); however, the prognostic value of HER2/*HER2* levels in Western patients with resectable GC remain elusive. To the best of our knowledge, only one study has documented the lack of correlation between HER2 expression level and prognosis in American patients with resectable GC (Fisher et al. 2014). The prognostic value of HER2/*HER2* levels in Western population needed to be further investigated.

In our work, *TP53, EGFR* and *PIK3CA* as classic driver genes (Ge et al. 2017) for gastric cancer, were the most commonly seen concomitant alterations in HER2-positive resectable GA patients. We found the presence of *TP53/EGFR/PIK3CA* mutations was not correlated with survival outcomes in HER2-positive resectable GA patients. The previous study also demonstrates that *TP53* mutations and *EGFR* amplification do not have an impact on progression-free survival of HER2-positive GC patients treated with trastuzumab (Lee et al. 2015). Furthermore, Harada et al. has reported *PIK3CA* mutations are not associated with DFS nor OS in patients with GC (Harada et al. 2016).

There are several limitations associated with our work. First, the prognostic value of *HER2* CN in HER2-positive resectable GA patients should be validated in a large prospective cohort. Second, intratumoral HER2/*HER2* heterogeneity was not evaluated in our study, which might result in bias of accurate assessment of HER2*/HER2* status due to the fact that intratumoral HER2/*HER2* heterogeneity is particularly significant in GC, ranging from 6 to 69% of HER2-postive GC patients and predicts unfavorable prognosis in HER2-positive GC (Grillo et al. 2016; Kaito et al. 2019). Further research is still needed to clarify the relevance of intratumoral HER2*/HER2* heterogeneity for the survival outcomes in HER2-positive resectable GA patients with *HER2* CN-high or CN-low.

In this study, the correlations between *HER2* CN and survival outcomes in HER2-positive resectable GA patients were investigated. Our findings demonstrated that *HER2* level was the independent predictor associated with DFS in HER2-positve resectable GA patients. Our work indicated *HER2* level can serve as a biomarker in predicting prognosis of patients with HER2-positive resectable GA.
